# Pure Primary Ovarian Squamous Cell Carcinoma Perforating the Rectum

**DOI:** 10.1155/2017/9793086

**Published:** 2017-02-20

**Authors:** Kazuya Mimura, Aiko Okada, Naotsugu Haraguchi, Kenjiro Sawada, Takuji Tomimatsu, Tadashi Kimura

**Affiliations:** ^1^Department of Obstetrics and Gynecology, Osaka University Graduate School of Medicine, Suita, Osaka, Japan; ^2^Gastroenterological Surgery, Osaka University Graduate School of Medicine, Suita, Osaka, Japan

## Abstract

Rectal perforation is uncommon in ovarian cancer, even in advanced stages. Pure primary ovarian squamous cell carcinoma is a very rare subtype of ovarian cancer and has not been reported to cause rectal perforation. A 50-year-old woman presented with rectal bleeding. Rectosigmoidoscopy suggested perforation of a pelvic tumor into the rectum. Abdominopelvic magnetic resonance imaging revealed a 9 cm heterogeneous mass in the pouch of Douglas. We performed complete cytoreduction, including an en-bloc resection of the tumor and rectosigmoid colon. Histopathology showed squamous cell carcinoma of the left ovary penetrating the rectal wall. A common symptom of rectal bleeding was caused by a very rare entity of ovarian cancer penetrating the rectal wall, but thorough evaluation led to its accurate diagnosis and appropriate treatment.

## 1. Introduction

Ovarian squamous cell carcinomas are uncommon, with most arising from malignant transformation of a mature cystic teratoma. Only a few appear in pure form [1, 2]. Most patients with ovarian cancer present at an advanced stage and have a poor prognosis. Invasion into the serosal and muscular layer of the sigmoid colon and rectum by advanced ovarian cancer is common, but colorectal perforation is an extremely rare presentation. Although fistulae communicating with the bladder, rectum, and intestine are reported in malignant transformation of mature cystic teratoma [3, 4], perforation by a pure primary ovarian squamous cell carcinoma has not been reported. We present a case of pure primary ovarian squamous cell carcinoma perforating the rectum.

## 2. Case Report

**Figure 1:**
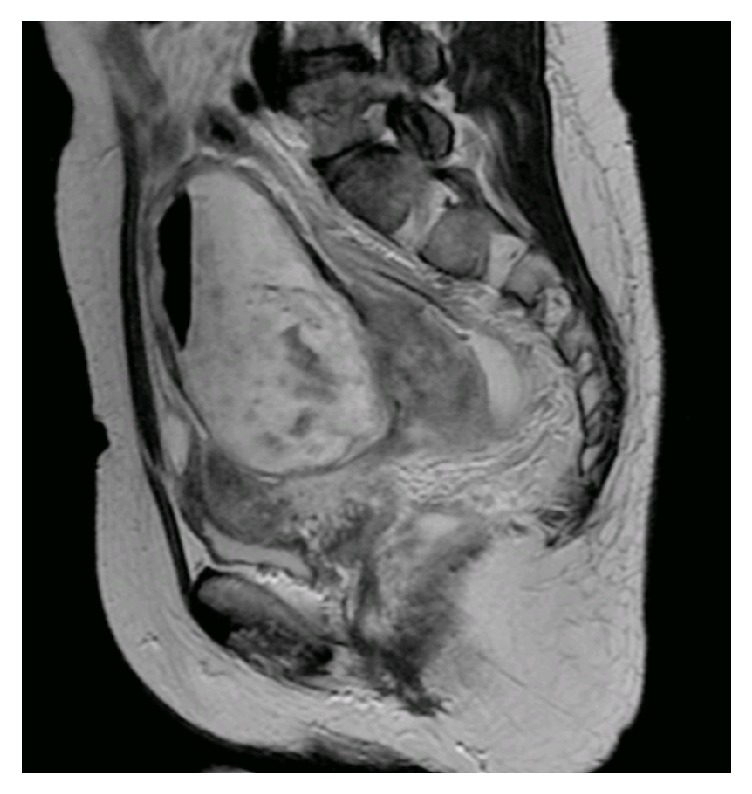
Abdominopelvic magnetic resonance imaging shows a complex mass containing a pocket of air in the pouch of Douglas.

A 50-year-old woman visited a gastroenterology outpatient clinic complaining of rectal bleeding for several days. There was no significant past history. Rectosigmoidoscopy suggested perforation of a pelvic tumor into the proximal part of the rectum. A biopsy specimen showed squamous cell carcinoma. She was then referred to our hospital. She had no fever and no lower abdominal tenderness on physical examination. Gynecological examination revealed a firm, fixed, fist-sized mass in the pouch of Douglas. Transvaginal ultrasonography revealed a 9 cm heterogeneous, solid mass. There was no free fluid. Laboratory findings included a normal blood count and biochemical parameters. Tumor marker values were normal: CA 125, 9 units/mL (normal: 0–65 units/mL); CA 19-9, 26 units/mL (0–40 units/mL); carcinoembryonic antigen, 3 ng/mL (0.4–3.1 ng/mL); and squamous cell carcinoma antigen, 1.5 ng/mL (0–2 ng/mL). Abdominopelvic magnetic resonance imaging indicated a complex mass containing a pocket of air, which appeared to be separate from the uterus and was seen to penetrate the proximal part of the rectum (Figure 1). Computed tomography revealed several slightly enlarged pelvic lymph nodes.

**Figure 2:**
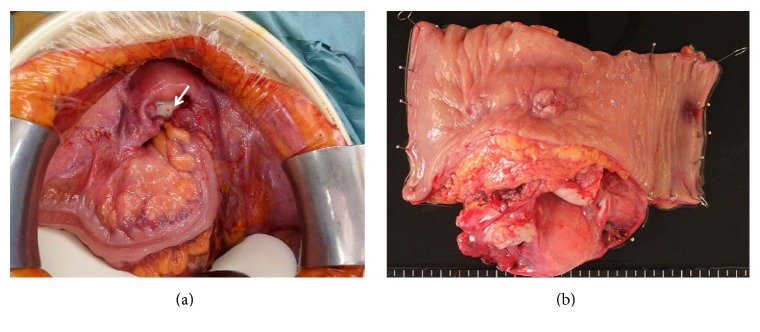
(a) At laparotomy, a left ovarian mass (arrow) was found adhering to the rectosigmoid colon. (b) Resected specimen revealing perforation into the rectum.

A laparotomy revealed a left ovarian mass adherent to the rectosigmoid colon (Figure 2(a)). The uterus and right adnexa were intact. Except for slight enlargement of pelvic lymph nodes, no signs of infection or other pathology were found in the rest of the abdomen. In order to resect the tumor and fistula tract, we performed an en-bloc resection of the uterus, adnexa, and rectosigmoid colon (Figure 2(b)). Frozen section examination of the specimen suggested ovarian squamous cell carcinoma, and we therefore performed infracolic omentectomy, pelvic and para-aortic lymphadenectomy, and ileostomy. The cytoreduction was complete.

**Figure 3:**
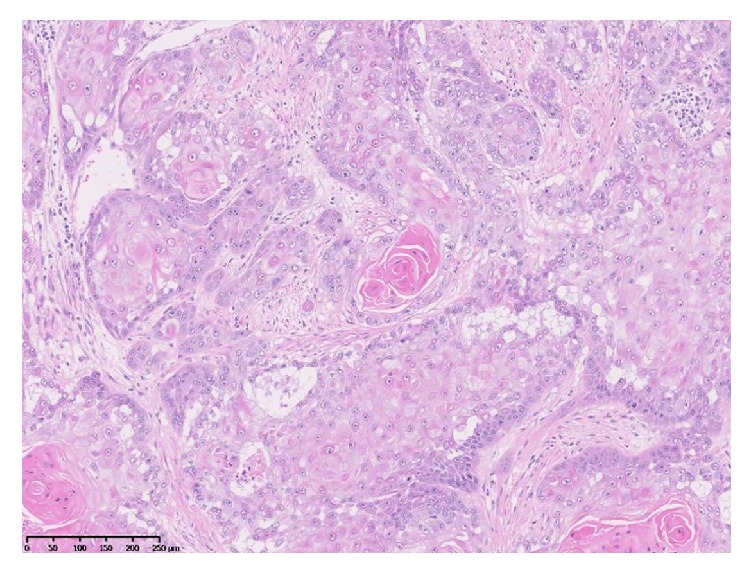
Microphotograph showing a well-differentiated squamous cell carcinoma (hematoxylin-eosin; ×100).

Pathological evaluation of the specimen confirmed the diagnosis of pure primary squamous cell carcinoma of the left ovary, secondarily penetrating through the rectal wall into the lumen. The International Federation of Gynecology and Obstetrics (FIGO) classification was stage IIB (Figure 3). There were no metastases to the lymph nodes or omentum. The patient had no postoperative complications and was discharged 18 days after the operation. Adjuvant chemotherapy (carboplatin and paclitaxel) was administered 42 days after the operation, and no recurrence was seen at the 7-month postoperative follow-up.

## 3. Discussion

Primary squamous cell carcinoma of the ovary is a very rare subtype of ovarian cancer, different from malignant transformation of a mature cystic teratoma, which may also contain squamous cells. Cases of ovarian cancer, including malignant transformation of mature cystic teratomas causing fistulae, involving the colon or rectum are extremely rare [4]. Here we describe the first case to be reported of pure primary ovarian squamous cell carcinoma perforating the rectum, which caused a common symptom of rectal bleeding.

Most ovarian squamous cell carcinomas arise from malignant transformation of mature cystic teratomas, but they have also been reported in association with endometriosis and in a pure form [1, 2]. In our patient, the histological diagnosis showed features of invasive squamous cells alone, without a surrounding teratoma containing elements from all three germ layers. One series has been reported of 37 cases of ovarian squamous cell carcinoma, 19 of which were associated with teratoma, seven with endometriosis, and 11 in the pure form [1]. Because few cases of pure squamous cell ovarian carcinoma have been reported, descriptions of the clinical and pathological features are sparse. Of the 11 cases of this entity reported by Pins et al., presentation occurred at a mean age of 56 years (range 27–73 years), with FIGO stages I, II, III, and IV reported in one, four, five, and one patients, respectively. The tumors ranged in size from 6 to 26 cm in greatest diameter and were usually solid with focal necrosis [1]. The prognosis for squamous ovarian carcinoma, independent of type, is poorer than that for ovarian epithelial cancers of all histologic types, according to most reports in the literature [2]. The overall survival of the patients with pure primary ovarian squamous cell carcinoma in the series reported by Pins et al. was not significantly different from that of the 19 patients with malignant teratoma [1]. The stage at diagnosis correlated best with survival but not with age at diagnosis, tumor diameter, or the presence or absence of necrosis. Five-year survival with advanced ovarian squamous cell carcinoma is better for patients with optimal debulking (76%) than that for patients whose surgical result was suboptimal (24.5%). The evidence is very limited, but adjuvant chemotherapy (mostly platinum-based) appeared to prolong survival in patients with stage III and IV disease compared with those who had surgery only [2, 5].

Perforation of ovarian cancer into the large bowel has been reported in only seven cases [6], including two cases of malignant transformation of mature cystic teratomas [3, 4]. Perforation into the rectosigmoid has also been reported with benign ovarian tumors, mainly mature cystic teratomas. In a review of 38 cases of fistulae associated with teratomas, 30 communicated with the urinary bladder and the others with the colon [7]. The exact factor in not clear, but these reports indicate that leakage of cyst fluid caused by torsion, trauma, malignant transformation, infection, or chronic pressure during labor leads to a fistula [8]. Malignant transformation has not always been shown as an essential factor in formation of a fistula [6]. In our case, there were no apparent factors described above, and tumor was not cystic. The exact pathophysiology is unknown, but severe adherence and chronic pressure could have caused the fistula in our case.

Bats et al. reported a case of ovarian carcinoma penetrating the rectosigmoid that was also associated with an abscess [6]. Our patient did not have an abscess, but we agree with their recommended surgical management that allows management of the fistula along with cytoreduction of the tumor [6]. In addition, chemotherapy is necessary because rectal involvement of an ovarian cancer is classified as at least FIGO stage II, although bowel resection or intra-abdominal infection may delay the start of chemotherapy. Such factors could contribute to a poor prognosis, but our patient is fortunate in that her rectal bleeding prompted a rapid diagnosis. The tumor was encapsulated and had not spread elsewhere in the pelvis, nor was significant inflammation present. We were able to perform an en-bloc resection of the tumor and complete debulking, which is a major prognosis factor in ovarian cancer. We were also able to start adjuvant chemotherapy without significant delay.

In conclusion, formation of a fistula by an ovarian cancer is extremely rare, with a few such cases reporting involved malignant transformation of a mature cystic teratoma. Pure primary ovarian squamous cell carcinoma was the cause of perforation in our patient. Rectal bleeding prompted the diagnosis, and timely, complete debulking surgery was performed. Thorough evaluation of a common sign such as rectal bleeding is crucial in determining the correct diagnosis and appropriate treatment.

## References

[1] Pins M. R., Young R. H., Daly W. J., Scully R. E. (1996). Primary squamous cell carcinoma of the ovary: report of 37 cases. *American Journal of Surgical Pathology*.

[2] Roxburgh P., Glasspool R. (2014). Squamous carcinoma of the ovary. *Current Oncology Reports*.

[3] Jewel K. L. (1977). Malignant teratoma with fistulous communication to the rectosigmoid. *American Journal of Proctology Gastroenterology and Colon and Rectal Surgery*.

[4] Mitui A. H., Fujita R., Sugata F., Kienebuchi M., Suzuki K., Sagawa F. (1983). A case of ovarian dermoid cyst with malignant transformation perforated into the rectosigmoid colon and small intestine. *Endoscopy*.

[5] Chen R.-J., Chen K.-Y., Chang T.-C., Sheu B.-C., Chow S.-N., Huang S.-C. (2008). Prognosis and treatment of squamous cell carcinoma from a mature cystic teratoma of the ovary. *Journal of the Formosan Medical Association*.

[6] Bats A.-S., Rockall A. G., Singh N., Reznek R. H., Jeyarajah A. (2010). Perforation of a malignant ovarian tumor into the recto-sigmoid colon. *Acta Obstetricia et Gynecologica Scandinavica*.

[7] Landmann D. D., Lewis R. W. (1988). Benign cystic ovarian teratoma with colorectal involvement—report of a case and review of the literature. *Diseases of the Colon & Rectum*.

[8] Cebesoy F. B., Baskonus I., Mete A., Kutlar I., Aybasti N. (2009). Benign ovarian dermoid cyst complicated with rectal fistula formation: an unusual case. *Archives of Gynecology and Obstetrics*.

